# Cardiac afferent activity modulates the expression of racial stereotypes

**DOI:** 10.1038/ncomms13854

**Published:** 2017-01-17

**Authors:** Ruben T. Azevedo, Sarah N. Garfinkel, Hugo D. Critchley, Manos Tsakiris

**Affiliations:** 1 Lab of Action & Body, Department of Psychology, Royal Holloway University of London, Egham TW20 0EX, UK; 2 The Warburg Institute, School of Advanced Study, University of London, Woburn Square, WC1H 0AB, UK; 3 Department of Psychiatry, Brighton and Sussex Medical School, Falmer BN1 9RR, UK; 4 Sackler Centre for Consciousness Science, University of Sussex, Falmer BN1 9RR, UK

## Abstract

Negative racial stereotypes tend to associate Black people with threat. This often leads to the misidentification of harmless objects as weapons held by a Black individual. Yet, little is known about how bodily states impact the expression of racial stereotyping. By tapping into the phasic activation of arterial baroreceptors, known to be associated with changes in the neural processing of fearful stimuli, we show activation of race-threat stereotypes synchronized with the cardiovascular cycle. Across two established tasks, stimuli depicting Black or White individuals were presented to coincide with either the cardiac systole or diastole. Results show increased race-driven misidentification of weapons during systole, when baroreceptor afferent firing is maximal, relative to diastole. Importantly, a third study examining the positive Black-athletic stereotypical association fails to demonstrate similar modulations by cardiac cycle. We identify a body–brain interaction wherein interoceptive cues can modulate threat appraisal and racially biased behaviour in context-dependent ways.

## Abstract (web summary)

Black individuals are racially stereotyped as threatening but how bodily signals may affect these misperceptions is not known. Here Azevedo and colleagues show that these race-driven responses are affected by the cardiac cycle, being more biased when arterial baroreceptor activation is maximal.

Recent statistics from USA reveal that Black Americans are more than twice as likely to be unarmed when killed during encounters with police as White people^1^. This is a striking example of how implicit stereotypes in physiologically arousing contexts may influence behaviour even among those who do not consciously endorse them. Notably, laboratory research has replicated this pattern among university students^2^ and police officers^3^,^4^, demonstrating that people are more likely to misidentify harmless objects as weapons when associated with a Black individual^5^ and are more prone to ‘shoot' unarmed Black targets^2^,^3^,^4^.

At the neural level, research has shown that the mere perception of Black individuals may trigger a pattern of neurophysiological responses consistent with rapid activation of the threat-signalling system, such as increased activity in the amygdala^6^ and enhanced startle potentiation^6^,^7^. Of interest, two studies have explored the effects of anxiety in the expression of Black-threat associations and found that high cortisol reactivity to stress may exacerbate vigilance for racial threat cues^8^ and reduce the ability to control stereotypic behaviour^9^. These studies hint to an influence of altered states of psycho-physiological arousal on racially biased behaviour, especially in relation to anxiety and altered patterns of physiological responses (for example, refs 10, 11, 12). However, the physiological afferent mechanisms that underpin the feedback of physiological states to the brain are typically overlooked. One contributing factor to this oversight is the relative difficulty in characterizing the mechanisms through which bodily states interact with perception and cognition, because it is methodologically challenging to disentangle afferent ‘body-to-brain' effects from efferent ‘brain-to-body' responses or arousal-related mental states^13^,^14^.

One prominent channel of body and brain communication is that conveyed by baroreceptors, pressure and stretch-sensitive receptors within the heart and surrounding arteries. Within each cardiac cycle, bursts of baroreceptor afferent activity encoding the strength and timing of each heartbeat are carried via the vagus and glossopharyngeal nerve afferents to the nucleus of the solitary tract^15^. This represents the major mechanism for fast and short-term regulation of blood pressure, through which increases in blood pressure trigger a parasympathetic-mediated reflex slowing of the following heartbeat and inducing sympatho-inhibitory peripheral vasodilatation^13^,^15^,^16^. Importantly, this is also the principal route that communicates to the brain the dynamic state of the heart, enabling the representation of cardiovascular arousal within viscerosensory brain regions, and influence ascending neuromodulator systems implicated in emotional and motivational behaviour^17^. Because arterial baroreceptors are activated by the arterial pulse pressure wave, their phasic discharge is maximal during and immediately after the cardiac systole, that is, when the blood is ejected from the heart, and minimal during cardiac diastole, that is, between heartbeats^18^,^19^. Capitalizing on this, recently developed experimental paradigms propose that it is possible to explore the impact of this interoceptive signal on psychological processes by timing the brief presentation of stimuli to the different phases of the cardiac cycle. In particular, it was observed that when certain categories of stimuli are presented during systole versus diastole, stimulus processing is enhanced or attenuated. This is typically taken as evidence that concurrent baroreceptor firing modulates brain–body interactions across a variety of psychological domains, including the processing of sensory^20^,^21^ and painful^18^,^22^ stimuli, and cognitive processes such as short-term^23^ and long-term^24^ memory. Of particular interest for the present study, previous research using this experimental approach found that the neural representation of certain emotional stimuli, such as fearful facial expressions, are co-modulated with baroreceptor signals^25^,^26^. In particular, the detection and appraisal of fearful faces is enhanced at systole and diminished at diastole, when the central representation of cardiovascular arousal is, respectively, maximal and minimal. At the neural level, these baroreceptor-related effects in the processing of threat stimuli are thought to be associated with changes in the engagement of brain systems involved in emotional processing and autonomic regulation including the amygdala and the insula^19^,^26^,^27^.

Thus, this experimental approach allows the presentation of stimuli in discrete yet short-term physiological contexts (baroreceptor activation or quiescence) to individuate the potential influence that heart-to-brain signals have on cognition and behaviour without inducing the subjective experience of altered cardiovascular arousal^17^. Here, we applied this strategy to investigate how the cardiac cycle modulates the processing (Study 1) and behavioural expression (Study 2) of racial stereotypes. On the basis of the known effects of cardiac cycle in fear processing^17^,^26^, we expected that racial stimuli presented at systole, compared with those presented at diastole, would be associated with greater racial bias when activating threat-related stereotypes (Studies 1 and 2) but not when associated with positive racial stereotypes (Study 3).

## Results

### Cardiac effects in weapons identification task

**Figure 1:**
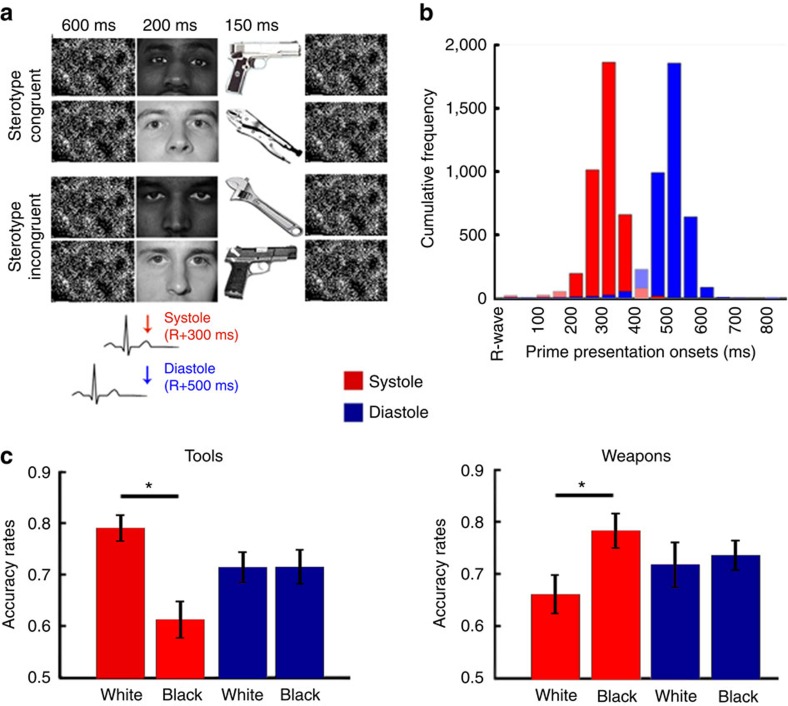
Study 1. (**a**) Graphical representation of the experimental procedure. In this adaptation of the WIT^5^, participants (*n*=30) had to discriminate between pictures (targets) of tools and weapons that were preceded by pictures (primes) of a White or a Black male face. Prime onset was synchronized with different phases of the cardiac cycle—systole or diastole—to investigate the modulatory role of cardiac afferent signalling on the expression of this stereotype. (**b**) Cumulative frequencies of stimuli onset (ms) relative to the R-wave peak for each cardiac cycle condition. Trials were then re-coded a-posteriori according to their onset time—stimuli presented between 200–400 ms were defined as systole and those between 450–800 ms defined as diastole. Excluded time intervals are displayed in faded colour. (**c**) Average accuracy rates (and standard errors) as a function of object type, prime and cardiac cycle. Results reveal that participants showed a relative enhancement of racial bias when primes were presented during the period of maximal baroreceptor activity (at systole) compared with reduced bias when it was minimal (at diastole).

In Study 1, we used the weapons identification task (WIT)^5^, a task designed to assess the implicit stereotypes of Black individuals as dangerous. Participants were required to make fast discriminations between images of handguns or tools primed with pictures of Black or White individuals' faces (Fig. 1). Because face perception activates semantic knowledge associated with each group category (for example, Black-threat), the presence of racial bias is typically reflected by improved identification of handguns and reduced identification of tools when preceded by Black primes compared with White primes^5^,^9^. We timed the presentation of the primes to coincide with the systolic (that is, ∼300 ms after the R-wave) or diastolic (that is, ∼500 ms after the R-wave) phases of the cardiac cycle. This showed that afferent cardiac activity influences the expression of racial bias, as demonstrated by the significant three-way interaction prime (Black/White) × object (tool/weapon) × cardiac cycle (systole/diastole) on a repeated measures ANOVA (*n*=30) on accuracy rates, *F*(1,29)=36.58, *P*<0.001, *η*^2^=0.56. The interaction prime × object was also significant, *F*(1,29)=24.69, *P*<0.001, *η*^2^=0.46, replicating the overall racial bias pattern typically observed in this task. Breaking down the interaction according to object type confirmed this effect in the identification of both tools (*F*(1,29)=19.36, *P*<0.001, *η*^2^=0.40) and weapons (*F*(1,29)=14.13, *P*=0.001, *η*^2^=0.32). Planned comparisons, using two-tailed paired *t*-tests (*n*=30), revealed better identification of weapons (*t*(1,29)=5.06, *P*<0.001, Cohen's *d*=0.92) after Black (versus White) primes and tools (*t*(1,29)=8.44, *P*<0.001, Cohen's *d*=1.54) after White (versus Black) primes at systole but not at diastole (*t*s<0.71, *P*s>0.45, Cohen's *d*s<0.13; Fig. 1). In other words, the processing of racial cues when the central signalling of cardiac activity was maximal (at systole), compared with when it was minimal (at diastole), resulted in an increase in stereotypic errors. Importantly, the cardiac cycle modulation of prime effects was dependent on semantic associations between the prime and the object, since Black faces presented at systole diminished the identification of tools yet improved the identification of weapons. We also carried out analyses on accuracy according to the signal detection theory (SDT)^28^ to explore how the ability to discriminate between tools and weapons (*d*′) and biases in response tendencies (*C*) varied according to prime type and cardiac cycle. For that, we calculated separate *d*′ and *C* indices as a function of prime and cardiac cycle and entered them into separate prime (Black/White) × cardiac cycle (systole/diastole) repeated measures ANOVAs (*n*=30). Analyses on *d*′ indices revealed that neither the interaction nor the main effects were significant (*F*s(1,29)<0.93, *P*s>0.34, *η*s^2^<0.031). Conversely, both the interaction prime × cardiac cycle (*F*(1,29)=42.01, *P*<0.001, *η*^2^=0.59) and main effect of race (*F*(1,29)=20.25, *P*<0.001, *η*^2^=0.41), but not the main effect of cardiac cycle (*F*(1,29)=0.06, *P*=0.82, *η*^2^<0.01), were found to have significant effects in *C*. Together, SDT results confirm that cardiac cycle did not influence object discrimination *per se* (*d*′) but enhanced the response bias (*C*) as a function of prime type.

According to theories of implicit cognition^29^,^30^, performance in this task is a product of different sub-processes that can be categorized as automatic, that is, the activation of racial associations, and controlled, that is, the ability to detect a correct response and overcome automatic associations. Using the performance dissociation procedure (PDP)^5^,^30^, we were able to compute independent estimates of these sub-processes and explore the effect of cardiac cycle in each of them. For that, indices of automatic and controlled processing were entered into separate repeated measures ANOVAs (*n*=30) with prime (Black/White) and cardiac cycle (systole/diastole) as within-subject factors. Interestingly, an effect of cardiac cycle was observed in automatic (*F*(1,29)=28.13, *P*<0.001, *η*^2^=0.49) but not in controlled processes (*F*(1,29)=0.91, *P*=0.35, *η*^2^=0.03), such that presenting primes at systole led to enhanced activation of racial stereotypes. In other words, as controlled behaviour failed due to the need to provide time-constrained decisions, automatic associations influenced behaviour, particularly when primes were presented at systole. Conversely, neither the interactions of prime × cardiac cycle (automatic: *F*(1,29)=0.35, *P*=0.56, *η*^2^=0.01; controlled: *F*(1,29)=2.0, *P*=0.17, *η*^2^=0.06) nor the main effects of prime (automatic: *F*(1,29)=2.29, *P*=0.14, *η*^2^=0.07; controlled: *F*(1,29)=1.16, *P*=0.29, *η*^2^=0.04) were significant, suggesting a similar influence of cardiac cycle on the automatic processing of Black-weapons and White-tools associations. It should be noted that the seemingly unexpected association White-tools is in fact a consequence of contextual contrast effects typically observed in evaluative priming tasks^31^. That is, given the predominant association Black-threat in this task, non-threatening objects are, by contextual contrast, associated with White faces leading to a response pattern reflecting the association White-thus-safety-thus-tool.

### Cardiac effects in first person shooter task

**Figure 2:**
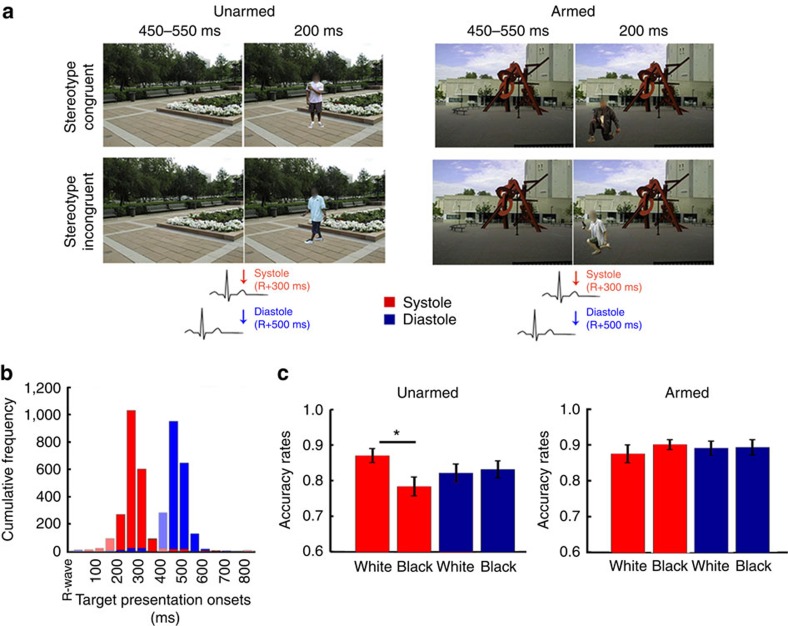
Study 2. (**a**) Graphical representation of the experimental procedure. In this adapted version of the FPST^2^, participants (*n*=30) were presented for a brief period (200 ms) with pictures of a White or Black male holding either a weapon or a harmless object (for example, phone, wallet) in his hand, and had to make the split-second decision of whether to ‘shoot' or ‘don't shoot' him, respectively. Crucially, we time-locked the presentation of the target to coincide either with the cardiac systole or the cardiac diastole. (**b**) Cumulative frequencies of stimuli onset (ms) relative to the R-wave peak for each cardiac cycle condition. Trials were then re-coded a-posteriori according to their onset time—stimuli presented between 200–400 ms were defined as systole and those between 450–800 ms defined as diastole. Excluded time intervals are displayed in faded colour. (**c**) Average accuracy rates (and s.e.) as a function of object type, race and cardiac cycle. Participants were more likely to ‘shoot' unarmed Black than unarmed White individuals when stimuli was perceived during systole compared to when perceived during diastole. For publication purposes, the images were blurred around the targets' faces to conceal the models' identity.

In Study 1 we investigated, for the first time, of how incoming afferent physiological signals may impact the modulation of racially biased behaviour. In Study 2, we extended these findings to situations requiring threat-related decision-making in complex scenarios. The first person shooter task (FPST)^2^ was designed to recreate the real-life scenario in which a police officer needs to make the split-second decision of whether to shoot or not a potentially armed individual. In each trial, participants are presented with a photo of a White or Black male holding either a gun or a harmless object in his hand. Participants are required to make the time-constrained decision to ‘shoot' or ‘not to shoot' based on whether the person is holding a gun or another object, respectively. Typically, participants are more likely to shoot unarmed Black individuals than unarmed White individuals^2^,^3^,^4^. By timing the onset of stimulus presentation to coincide with cardiac systole or diastole, we tested the influence of afferent cardiovascular information on the expression of racial bias (Fig. 2). Similarly to Study 1, the three-way interaction object (weapon/no weapon) × race (Black/White) × cardiac cycle (systole/diastole) on the repeated measures ANOVA (*n*=30) on accuracy rates was found to be significant, *F*(1,29)=5.40, *P*=0.027, *η*^2^=0.16, confirming the effect of cardiac cycle on racially biased decision-making. The prime × object interaction, *F*(1,29)=4.38, *P*=0.045, *η*^2^=0.13, was also significant, replicating the overall racial bias pattern typically observed in this task. Separate analysis on the cardiac cycle × race interactions for each object type revealed that the phase of the cardiac cycle modulated racially biased responses to unarmed targets, *F*(1,29)=6.24, *P*=0.018, *η*^2^=0.18 (main effect of race: *F*(1,29)=5.21, *P*=0.030, *η*^2^=0.15; main effect of cardiac cycle: *F*(1,29)<0.01, *P*=0.98, *η*^2^<0.01). Planned comparisons, using two-tailed paired *t*-tests (*n*=30), further revealed that participants ‘shot' more often unarmed Black targets than White targets when the stimuli were presented at systole, *t*(1,29)=3.02, *P*=0.015, Cohen's *d*=0.55, but not at diastole, *t*(1,29)=−0.46, *P*=0.71, Cohen's *d*=0.08 (Fig. 2). Thus, in line with Study 1, presenting stimuli during periods of enhanced representation of cardiac activity was associated with increased salience of racial cues, leading to an increased likelihood of shooting ‘unarmed' Black individuals. However, cardiac cycle did not modulate racial bias on armed trials, *F*(1,29)=1.08, *P*=0.31, *η*^2^=0.04 (main effect of race: *F*(1,29)=0.80, *P*=0.38, *η*^2^=0.03; main effect of cardiac cycle: *F*(1,29)=0.02, *P*=0.88, *η*^2^<0.01). While we had predicted cardiac cycle to modulate behavioural responses to armed targets as well, these results are not surprising as this task may be less sensitive to the detection of cardiac cycle effects over racial cues in the ‘armed' conditions for at least two reasons: (i) task instructions encourage participants to provide a ‘shoot' response (see ‘Methods' section) leading to generalized higher accuracy in ‘armed' trials. Cardiac cycle effects may be less likely to be observed when performance approaches celling levels; (ii) in these trials an objectively threatening stimulus is present independently of the target's race which may further hinder the observation of the expected biases in activation of racial threat stereotypes by cardiac cycle.

We also carried out SDT analyses by submitting *d*′ and *C* indices to separate race (Black/White) × cardiac cycle (systole/diastole) repeated measures ANOVAs (*n*=30). Results on *d*′ indices revealed that while neither race nor cardiac cycle influenced object discrimination (Fs(1,29)<1.03, Ps>0.32, *η*s^2^<0.034), the interaction was significant (Fs(1,29)=4.72, *Ps*=0.038, *η*s^2^<0.14), suggesting different ability to discriminate objects held by a Black or White persons as a function of cardiac cycle. However, *post hoc* analyses, using two-tailed paired *t*-tests (*n*=30), failed to identify differences between conditions (*t*s(1,29)<2.04, Ps>0.11, Cohen's ds<0.38) and therefore these results should be interpreted with caution. No significant interaction nor main effects in response criteria as a function of race and cardiac cycle (Fs(1,29)<3.02, Ps>0.093, *η*s^2^<0.10) was found. The apparent contrasting results between ‘*C*' and main analyses in accuracy rates should be explained by the fact that, as discussed above, performance approached celling levels in the ‘armed' conditions. That is, while *d*′ and *C* indices simultaneously considers decisions in both ‘armed' and ‘unarmed' conditions, baroreceptor effects were only found to modulate race bias in ‘unarmed' trials.

In conditions of relative ambiguity and/or when acting under pressure, such as when a police officer needs to make a split-second decision of whether to shoot or not, contextual cues may have a strong influence on judgments and behaviour. Although seemingly irrelevant for the discrimination between a weapon and a harmless object, social (for example, Black versus White individuals) and contextual (for example, dark alley versus rich neighbourhood) cues may shift perceptual judgments and increase the probability of perceiving the situation as threating^32^. Likewise, emotional states of anger^33^, anxiety and sustained states of psycho-physiological arousal enhance vigilance to threat-related stimuli^34^ and influence racially biased behaviour^8^,^9^. We extend these observations by demonstrating the discrete power of fine-grained dynamic communication of cardiovascular arousal wherein preconscious fluctuations in interoceptive afferent activity amplify the impact of racial cues in biasing threat-related behaviour. Importantly, our results identify for the first time, bottom-up body-to-brain correlates for racially biased behaviour. This represents a clear departure from the traditional approaches that do not differentiate afferent from efferent effects. That is, past studies measured physiological responses to racial stimuli or used anxiety enhancing techniques that, although eliciting states of bodily arousal^8^,^9^, do not disentangle the discrete modulatory effects of afferent bodily signals from that of anxiety-related cognitive processing. In contrast, our approach in which the critical physiological conditions are likely to reflect rapid neurally signalled cardiovascular states, allowed us to observe within-participant fluctuations in the expression of racial bias and explore the influence that body-to-brain signals may have on social threat processing and intergroup biases.

### Cardiac effects in sport-fruits identification task

**Figure 3:**
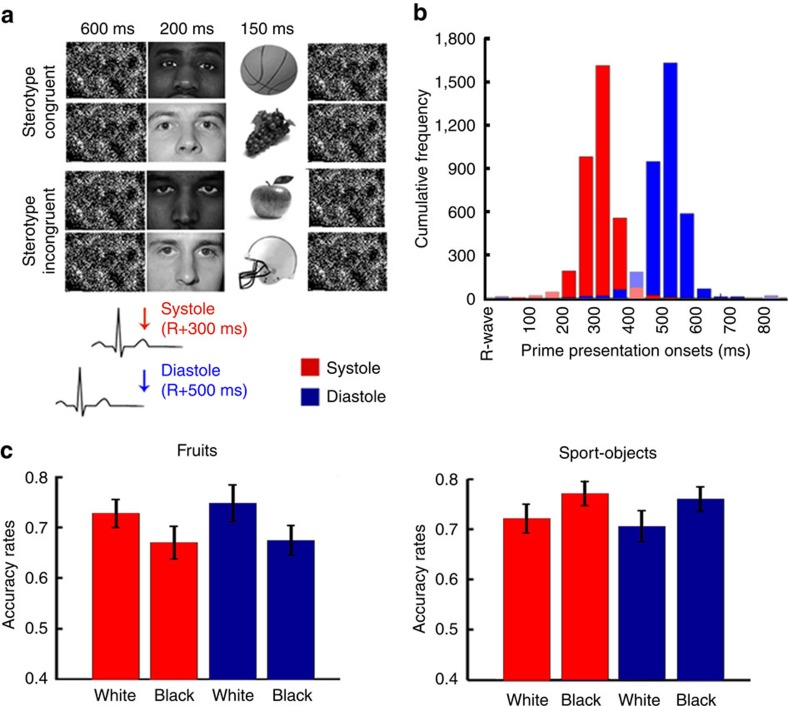
Study 3. (**a**) Graphical representation of the experimental procedure. The sport-fruit identification task (SFIT)^34^ is a modified version of the experimental paradigm used in Study 1 in which participants (*n*=30) need to discriminate between pictures (targets) of fruits and sport-objects that are preceded by pictures (primes) of a White or a Black male face. As in Study 1, we synchronized prime onset to coincide with the cardiac systole or diastole. (**b**) Cumulative frequencies of stimuli onset (ms) relative to the R-wave peak for each cardiac cycle condition. Trials were then re-coded a-posteriori according to their onset time—stimuli presented between 200–400 ms were defined as systole and those between 450–800 ms defined as diastole. Excluded time intervals are displayed in lighter shades. (**c**) Average accuracy rates (and standard errors) as a function of object type, race and cardiac cycle. Primes successfully induced response bias as reflected by the prime × object interaction. In particular, we observed greater accuracy in the identification of sport-objects preceded by Black (versus White) faces and improved identification of fruits preceded by White (versus Black) faces. However, in contrast with Study 1, this effect was not modulated by cardiac cycle. Together with the results from Study 1, Study 3 shows that cardiac cycle effects in the processing of racial cues are context dependent, such that the salient negative association Black-threat, but not the positive Black-athletic stereotype, is modulated by baroreceptor-mediated effects of interoceptive feedback.

However, racial bias toward Black individuals is not uniformly negative (for example, associated with threat). Depending on the context, different stereotypes may be activated. In Study 3, we investigated if the reported effects of cardiovascular interoceptive signals can be generalized to positive racial associations portraying Black individuals as athletic^35^,^36^. The sport-fruits identification task^35^ is a modified version of the WIT in which participants were required to discriminate between pictures of fruits and sport-objects preceded by pictures of White or Black faces. Once more, we timed the presentation of primes to coincide with the systolic or diastolic phases of the cardiac cycle (Fig. 3). While the three-way interaction prime (Black/White) × object (fruits/sport-objects) × cardiac cycle (systole/diastole) of the repeated measures ANOVA (*n*=30) was not significant (*F*(1,29)=0.30, *P*=0.59, *η*^2^=0.01), the two-way prime × object was significant (*F*(1,29)=11.36, *P*=0.002, *η*^2^=0.28), replicating the typically observed bias in object identification by racial primes. Breaking down the interaction according to object type confirmed improved identification of sport-objects (*F*(1,29)=6.26, *P*=0.018, *η*^2^=0.18) preceded by Black (versus White) faces and increased accuracy in the discrimination of fruits (*F*(1,29)=8.24, *P*=0.008, *η*^2^=0.22) preceded by White (versus Black) faces. However, cardiac cycle did not modulate this bias in either case (Fs(1,29)<1.05, Ps>0.31, Zss<0.035; Fig. 3). Analyses of *d*′ revealed that neither the cardiac cycle × prime interaction (repeated measures ANOVA, *n*=30) nor the main effects were significant (Fs(1,29)<0.51, Ps>0.48, *η*s^2^<0.02). Conversely, an effect of prime (*F*(1,29)=9.38, *P*=0.005, *η*^2^=0.24) could be observed in response bias, showing that Black primes induced a greater tendency to provide the sport-object response. Neither the main effect of cardiac cycle (*F*(1,29)=2.1, *P*=0.149, *η*^2^=0.07) nor the interaction were significant (*F*(1,29)=1.18, *P*=0.29, *η*^2^=0.04), confirming that cardiac cycle did not modulate racial bias. Thus, together these studies show that cardiac cycle effects on the expression of implicit racial bias is not the same across all social stereotypes (see Supplementary Note 1 for a statistical comparison between Study 1 and Study 3). Importantly, the fact that primes in Study 3 induced a robust behavioural bias confirms that the results reported in Studies 1 and 2 do not reflect a general baroreceptor effect over pure perceptual or inhibitory processes^37^,^38^ but instead depend on the contextual and semantic content of the activated associations.

Black-threat and Black-athletic stereotypes differ in several ways, but perhaps, most significantly they differ in terms of valence and salience of the associated emotions. This might be the key for the different results observed. The impact of incoming interoceptive information on the activation of semantic and affective reactions should depend on the relevance that bodily states of arousal have in the expression of the associated emotion and contingent behavioural response. In conditions of high vigilance, such as when discriminating between a weapon and a harmless object, the feedback of bodily states may be particularly informative in guiding behaviour. Conversely the positive, and presumably motivationally less relevant, stereotype Black-athletic may rely to a lesser extent on bodily information. This is in line with previous research showing preferential baroreceptor-related modulation in the processing of certain emotional categories, notably fear^17^,^26^. We suggest that those forms of bias associated with strong emotional, or motivational, content may be particularly sensitive to feedback of cardiovascular arousal, as revealed by the potentiation of effects within the cardiac cycle.

## Discussion

There is a growing consensus that the continuous dynamic cortical representation of internal bodily states, such as the ones conveyed by cardiac afferent signals, shape emotions and set the foundations for a sense of self as the basis for subjective experience^13^,^14^,^39^,^40^,^41^,^42^,^43^. It is also believed that preconscious short-term fluctuations in baroreceptor activity provide the physiological context through which body–brain effects can modulate the processing of, and subsequent neurophysiological responses to, sensory^18^,^20^,^21^,^22^ and emotional^25^,^26^ stimuli. Here, by synchronizing stimuli presentation to this signal we were able to investigate, without inducing sustained states of psycho-physiological arousal, how the processing of complex social stimuli and associated behavioural consequences may change in conditions in which the representation of cardiac activity in the brain is maximal compared with when it is minimal^17^. Although causal relationships between baroreceptor firing and racially biased behaviour are difficult to confirm empirically, our results suggest that this interoceptive mechanism may constitute a channel for body-to-brain influences in the processing of motivationally salient social stimuli. In particular, we propose that, in the context of alertness to threat-signalling stimuli, heightened representation of cardiac signals in the brain may enhance the salience of social cues and promote the expression of negative racial stereotypes.

Arterial baroreceptors signals are carried by cranial nerves X and XI directly to the nucleus of the solitary tract, which in turn has proximate connections with regions such as the thalamus and the amygdala. The observation of the enhanced activation of a negative racial stereotype by cardiac afferent information is likely to be underpinned by functional changes in these regions and in particular within the amygdala, a centre already implicated in baroreceptor effects on the processing of salient^19^ and fear stimuli^17^,^26^. Moreover, specific amygdala nuclei play central roles in coding the salience and associative relevance of perceptual stimuli^44^,^45^,^46^, particularly in the context of threat processing, including the modulation of attention to fear-relevant stimuli, fear conditioning and regulation of autonomic states of fear and anxiety^47^,^48^. Amygdala nuclei are also engaged in out-group face perception, threat detection and learning of social fears^49^,^50^. Interestingly however, amygdala activity to racial out-group stimuli is modulated by the social-cognitive goals and the presence of threat-related cues^51^,^52^. Thus, the observed exaggeration of racial biases in a highly salient, threat-related context is likely to be mediated through modulation of amygdala responsiveness by interoceptive signalling originating in phasic arterial baroreceptor activity. In other words, in the context of alertness to threat-signalling stimuli, amygdala responses to relevant environmental cues may be modulated by the strength and quality of the cardiac signals.

Another possible parallel mechanism relates to the known inhibitory effect of cardiovascular baroreceptor firing on activity within higher order cortical regions including dorsolateral prefrontal cortex, via influences on thalamocortical gating, reducing the ability to inhibit prepotent responses^38^. The implication would be that baroreceptor activity would increase the expression of racial stereotypes by compromising the ability to overcome undesirable automatic responses. While these mechanisms are not mutually exclusive, two pieces of evidence favour the tentative explanation of enhanced activation of semantic associations mediated by amygdala. First, our PDP analyses pinpoint an effect over automatic but not controlled processes. Second, Black-athletic stereotypes also activated prepotent responses but these were not modulated by cardiac timing. Future studies may help to elucidate this further.

It could be tempting to interpret the pattern of results observed in studies 1 and 2 as evidence for extinguished bias at diastole but such interpretation should be done with caution. The experimental approach used here compares relative contributions of the central representation of cardiovascular arousal in the processing of certain social stimuli by tapping into the extremes of ongoing fluctuations of baroreceptor activity which co-vary with the cardiac cycle. Past research on cardiac timing argues for the relative nature of baroreceptor effects (for example, refs 26, 38), that is, a relative enhancement of fear processing at systole and an inhibition at diastole^26^. Garfinkel *et al*.^26^ also showed that the relative inhibition of fear processing at diastole was particularly evident among low anxiety individuals, and suggested that the diminished inhibition of threat processing in anxious individuals may reflect a mechanism contributing to the exacerbation of fear signals in anxiety. While we have not measured anxiety levels among our participants, we speculate that a similar mechanism may be at play here. Inhibition of racial bias at diastole may reflect a mechanism through which the signalling of threat by members of a social group is attenuated. It is known that anxiety and sustained states of arousal impact and exacerbate intergroup biases^10^,^11^,^12^. Without inhibitory mechanisms such as the one proposed here racial associations would be more likely to escalate over time, promoting anxiety and reducing the ability to control racial bias. This account is consistent with current amygdala-centred models of learning and expression of aversive social associations^49^,^50^, according to which the amygdala nuclei serve two main functions: (i) form and store associations between somatosensory states and representations of particular stimuli (for example, Black individuals); (ii) and mediate the physiological responses to the associated stimuli. Our results build up on this by suggesting the influence of the relative strength or weakness of afferent cardiac sensations in the flexible enhancement and suppression of amygdala responsiveness to conditioned stimuli. However, further research is needed to determine how interoceptive signals contribute to the promotion of anxiety in intergroup interactions.

Lastly, while the present research focused on threat-related processes, we have no reason to believe that these associations between cardiac cycle and the potentiation of social associations are specific to race or fear processing. Indeed, we predict the extension of these effects to other social groups/stereotypes associated with substantial affective or motivational relevance. We suggest this may be an important body-to-brain mechanism affecting the processing of salient social signals and subsequent actions accordingly. In line with current accounts highlighting the predictive inferential nature of the brain, the (mis)perception of a gun or an object is a consequence of prediction-based perception shaped by previous social associations and concurrent bodily signals (53). Future research should investigate the neural underpinnings of these effects, as well as the functional correlates and the apparent inhibition of negative racial bias in conditions of quiescent baroreceptor firing. To the extent that this body-to-brain mechanism can be used as an intervention to accelerate unlearning of fear or threat-related stimuli, our results may have a substantial impact in the development of training interventions in professional settings where negative stereotypes may play a crucial role.

## Methods

### Participants

In Study 1, data was collected from 32 (24 females; mean age=21.8, s.d.=4.4) White healthy volunteers. Data from two participants was excluded from analyses due to incorrect use of response keys or noisy electrocardiogram (ECG) signal. Thus, the final sample comprised 30 (22 females; mean age=22, s.d.=4.5) participants. In Study 2, data was collected from 35 (26 females; mean age=22.0, s.d.=4.6) non overlapping White healthy volunteers. Data from five participants was excluded due to reported difficulties in discriminating the different types of stimuli (see Supplementary Methods 1). The final data set comprised 30 (21 females; mean age=22.2, s.d.=3.6) participants. In Study 3, the sample comprised 30 new healthy volunteers (67% White; 30% Asian; 7% mix White-Asian; 25 females; mean age=21.6, s.d.=3.7; see Supplementary Fig. 1 for control analyses related to participants' race). Data from one additional participant (White female) was excluded from analyses due to suspicion of incorrect use of the response keys (accuracy rates between 9 and 49%). All participants gave written informed consent. All studies were approved by the Departmental Ethics Committee, Department of Psychology, Royal Holloway University of London.

### ECG recording

Three disposable ECG electrodes were placed in a modified lead I chest configuration: two electrodes were positioned underneath the left and right collarbone and another on the participant's lower back on the left side. The ECG signal was recorded with a Powerlab 8/35 (Powerlab, ADInstruments, http://www.adinstruments.com/) using LAbchart 8 Pro software. The sampling rate was 1,000 Hz and a hardware band-pass filter (Bio Amp 132) between 0.3 and 1,000 Hz was applied. Heartbeats were detected online with a hardware based function (fast output response), which identifies the ECG R-wave with a delay smaller than 1 ms (www.adinstruments.com/) by detecting when the amplitude exceeds an individually defined threshold. Internal lab reports using this method confirm that the software presenting the stimuli receives the transistor-transistor logic (TTL) pulse signalling a heartbeat and is able to process it within <2 ms.

Because our designs included the presentation of a mask/background image up to 600 ms before the prime/target picture onset, and therefore previous to the reference R-wave, the timings of occurrence of the reference heartbeat had to be predicted. This was achieved with a custom-made software, implemented in Matlab (Matworks, http://uk.mathworks.com/), based on the timing of the three preceding R-waves. The reliability of this predictive method was tested by measuring a-posteriori the time distance between stimuli onset and the previous R-wave. The histograms of cumulative frequencies of stimuli onset (ms) relative to the R-wave peak for each cardiac cycle condition are displayed in Figs 1, 2, 3b. Despite the high predictive power of our method, some overlap between systolic and diastolic trials was present. To address this issue, trials were re-coded according to the actual stimulus onset. Following previous literature, the systolic period was defined as 200–400 ms after the R-wave (for example, refs 18, 26, 54), which is the period of maximal representation of baroreceptor afferent activity and when cardiac cycles effects are typically observed. Diastole, the remaining period of the cardiac cycle, was defined as 450–800 ms after the R-wave. Stimuli presented between 400 and 450 ms were excluded as the 400–450 ms window represent a challenge for the accurate classification of systole/diastole. For example, physiologically, the presentation of a stimulus at 395 ms should not be too different from another presented at 405 ms but with this classification method they are expected to have opposite effects. Trials in which stimulus was presented after 800 ms or between 0–200 ms after the R-wave were also excluded to avoid the possibility that target onset (which follows immediately primes) partially overlapped with the systolic period. Across the three studies percentages of excluded stimuli were smaller than 10.5%.

### Study 1

*Weapons identification task*. Stimuli consisted of black and white photos adapted from^5^,^55^. Primes were photos of a Black or White male face with neutral expression^55^. Twelve Black and 12 White models were used. Target stimuli consisted of a photo of a handgun or a tool (for example, wrench, pliers). Six different tools and handguns were used. Presentation timings were adapted to allow synchronization with the cardiac cycle. The prime was presented for 200 ms with the onset timed to coincide either with the end of the systolic phase (that is, ∼300 ms after the R-wave) or during the diastolic period (that is, ∼500 ms after the R-wave). Immediately after, the target was presented for 150 ms. These presentation timings were adopted so that primes were presented at systole or diastole and targets onsets occurred outside the critical systolic period. This allowed us to investigate the effects of baroreceptor activity in the activation of racial stereotypes and not in the processing or response to threat-related objects. Before the prime and after the target a mask was presented for 600 and 300 ms, respectively, independently of cardiac cycle condition. A fixation cross in the centre of the screen was displayed between trials. inter-trial intervals consisted in the (variable) time period necessary to detect four heartbeats and predict the timing of the subsequent cardiac cycle during which the stimuli would be presented. Participants' task was to press the ‘i' key, with their right hand, if the target was a weapon and the ‘e' key, with their left hand, if the target was a tool. They were instructed to be as fast and accurate as possible and were told that failures to provide an answer within 500 ms would lead to a feedback warning ‘Please try to be quicker'. Nevertheless, to allow reaction times analyses, response latencies up to 650 ms were collected. Response keys associated with weapons and tools were not counterbalanced across participants. This may not be ideal, as it may lead to an unbalanced use of the dominant hand to identify one of the targets and influence the ability to quickly respond to weapons versus tools. Nevertheless, given the within-participant-and-target nature of our effects of interest, it should have no influence on the observed behavioural patterns related to cardiac cycle effects. A total of 264 trials, 33 per condition, were presented in a fully randomize order. Half-way through the task, participants were asked to rest for at least two minutes. In order to familiarize participants with the stimuli and task procedures, two practice blocks were administered. In the first practice block, comprised of 12 trials, no primes were presented and no time limit was given for target identification. The second practice block consisted of 12 trials in which primes and the response time limit were introduced.

Accuracy rates (that is, on-time correct responses divided by the number of trials) were analysed both through standard analyses of variance, that is, prime (Black/White) × object (tool/weapon) × cardiac cycle (systole/diastole) ANOVA, and according to SDT. In the SDT analyses, separate *d*′ and *C* indices were estimated as a function of prime and cardiac cycle and entered into separate prime (Black/White) × cardiac cycle (systole/diastole) ANOVAs. ‘Hit rates' were defined as the percentage of correct responses to weapons and ‘false alarms' as the percentage of incorrect responses to tools.

We have also used the PDP to estimate the contribution of automatic (PDP-automatic) and controlled (PDP-control) processes to performance in this task. PDP-control indices were estimated separately for Black and White prime conditions by subtracting error rates on stereotype incongruent trials (Black-Tool and White-Weapon, respectively) from accuracy on stereotype congruent trials (Black-Weapons and White-Tool, respectively). PDP-automatic indices were computed by dividing error rates on stereotype incongruent trials by the respective reciprocal of PD-control scores^5^. Cardiac cycle effects were explored by estimating these indices separately for trials at systole and diastole.

All *P* values from *post hoc* comparisons were corrected using the Newman–Keuls method. Analyses of reaction times revealed equivalent results (Supplementary Fig. 2). Please refer to online Supplementary Information for: (i) analyses referring to participant's individual interoceptive accuracy^56^ (Supplementary Note 2) and (ii) a Figure showing the distribution of stereotypic errors across participants at the different phases of the cardiac cycle (Supplementary Fig. 3a).

### Study 2

*First person shooter task*. Stimuli consisted of pictures taken from^2^,^3^ depicting White and Black male individuals holding a handgun or another object (for example, mobile phone, wallet). Participants were instructed to act as a police officer and ‘shoot' the target by pressing the key ‘i', with their right hand, if he had a gun and ‘not to shoot' him by pressing the ‘e' key, with their left hand, if he did not have a gun. Response keys associated with weapons and tools were not counterbalanced across participants. Each trial began with the presentation of a background image (for example, a street, a park) for 400–500 ms after which the target image was superimposed for 200 ms. The presentation of the target was time-locked to coincide with systole (that is, ∼300 ms after the R-wave) or diastole (that is, ∼500 ms after the R-wave). Unlike previous research with this task, we did not present a random number of backgrounds before the target onset due to time constrains linked with the synchronization with heart timings. A fixation cross was present on the centre of the screen for the entire inter-trial-interval. The task comprised a total of 180 trials (23 per condition) but only 148 different pictures were used, meaning that 32 pictures, randomly selected for each participant, were presented twice. Stimuli to be presented at systole and diastole were also randomly selected for each participant. Half-way through the task, participants were asked to rest for at least two minutes. The task included two blocks of practice trials, comprised by 12 trials each. The images presented were different from those used in the experimental task. No time limit was given for responses in the first practice block. In the second block the response time limit was introduced.

Participants were told that if they failed to make a key press within 650 ms they would receive the feedback ‘Please try to be quicker'. Nonetheless, to allow reaction times analyses, responses up to 1,000 ms after target onset were collected. Participants were awarded and deducted points on the basis of their performance (shooting armed targets: +10 points; not shooting unarmed targets: +5 points; shooting unarmed targets: −20 points; not shooting armed targets: −40 points; failing to give an answer within 650 ms: −10 points). These payoffs are implemented to partially simulate the payoff matrix experienced by police officers on the street^2^. They were also told that the 3 best players from the entire pool of participants would be awarded a reward (£20–first place; £10–second place; £5–third place). Accuracy feedback and updated total of points was provided after each trial (see Supplementary Methods 1 for further details).

Accuracy rates (that is, the ratio between on-time correct responses and the number of valid trials) were analysed both through standard analyses of variance and according to SDT. Here, ‘hit rates' were defined as the percentage of correct responses in the ‘shoot' condition and ‘false alarms' as the percentage of errors in the ‘don't shoot' condition. *P* values from *post hoc* comparisons were corrected using the Newman–Keuls method.

Participants also carried out the Implicit Association Test^55^ to measure how performance in the FPST relates to general implicit attitudes towards Black individuals (See Supplementary Note 3). Please refer to online Supplementary Information for: (i) analyses of reaction times (Supplementary Fig. 4) and (ii) a Figure showing the distribution of stereotypic errors across participants at the different phases of the cardiac cycle (Supplementary Fig. 3B)

### Study 3

*Sport-fruits identification task*. Procedures and experimental design were equivalent to that of Study 1 with the exception of the target stimuli used. Here, participants had to discriminate between pictures of fruits (for example, apple, grapes, cherries) and sport-objects (for example, basketball ball, weights, skateboard)^35^. This task taps into the stereotype of Black individuals as athletic^35^,^36^. Ten black and white pictures of each stimuli category were used. The ‘e' and ‘i' keys were assigned to fruits and sport-related objects, respectively.

As in Study 1, accuracy rates (that is, on-time correct responses divided by the number of trials) were analysed both through standard analyses of variance and according to SDT. In the SDT analyses, ‘hit rates' were defined as the percentage of correct responses to sport-objects and ‘false alarms' as the percentage of incorrect responses to fruits.

Analyses of reaction times revealed equivalent results (Supplementary Fig. 5). Please refer to online Supplementary Information for a Figure showing the distribution of stereotypic errors across participants at the different phases of the cardiac cycle (Supplementary Fig. 3C).

### Data availability

Data reported in this manuscript are available on request from the authors.

## Additional information

**How to cite this article:** Azevedo, R. T. *et al*. Cardiac afferent activity modulates the expression of racial stereotypes. *Nat. Commun.* **8,** 13854 doi: 10.1038/ncomms13854 (2017).

**Publisher's note**: Springer Nature remains neutral with regard to jurisdictional claims in published maps and institutional affiliations.

## Supplementary Material

Supplementary Information Supplementary Figures, Supplementary Notes, Supplementary Methods and Supplementary References
