# Supplementary material for: Cardiac afferent activity modulates the expression of racial stereotypes
Source: Nat Commun. 2017 Jan 17;8:13854. doi: 10.1038/ncomms13854 (PMC5247580; doi:10.1038/ncomms13854)
Supplement: Supplementary Information — Supplementary Figures, Supplementary Notes, Supplementary Methods and Supplementary References [file ncomms13854-s1.pdf]

Supplementary Information

Supplementary Figure 1

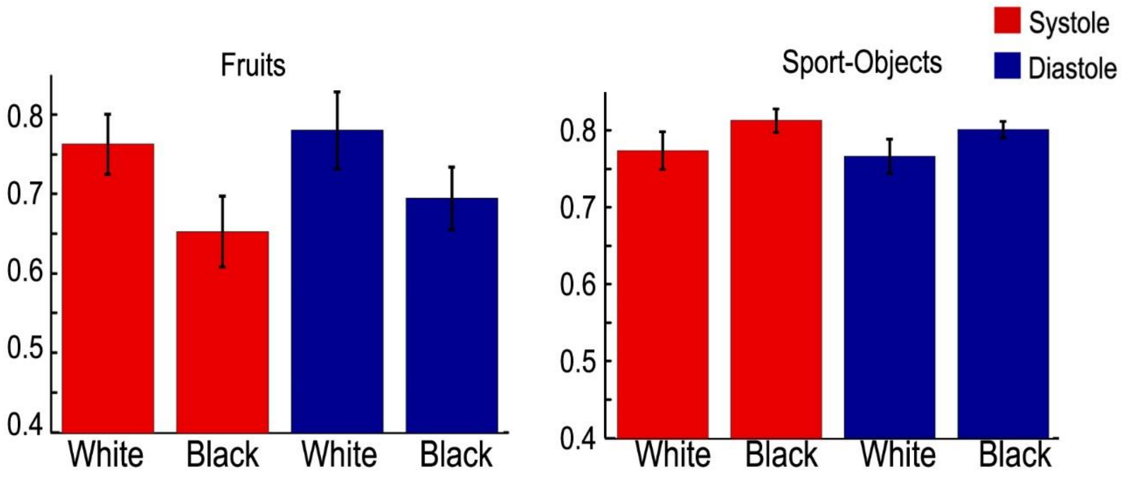

Supplementary Figure 1. Average accuracy (SEM) in the SFIT **(Study 3)** as a function of prime race, object type and cardiac cycle for white participants only. The recruitment of non-White volunteers (e.g. Asian, mixed-Asian), here referred to as non-Black, is standard practice in studies of this nature (e.g. 1-4). However, to ensure that the participant's race had no influence in the pattern of results observed we carried out analyses on average accuracy rates considering only the 19 white participants. Results are in accordance with those observed in the analyses of the full sample. In specific, while the Prime (Black/White) x Object (Fruit/Sport-Object) x Cardiac Cycle (Systole/Diastole) interaction was not significant ($F(1,18)=0.04$, $p=0.84$, $\eta 2<0.01$) the critical Prime X Object interaction reached significance ($F(1,18)=10.04$, $p=0.005$, $\eta 2=0.36$ (repeated measures ANOVA, n=19). We have also reanalysed accuracy data on the entire dataset including participants' race as a between subjects factor in a Race (White/non-Black) x Prime (Black/White) x Object (Fruit/Sport-Object) x Cardiac Cycle (Systole/Diastole) ANOVA (n=30). Race did not interact with any factor ($Fs(1,18)<1.41$, $ps>0.24$, $\eta 2<0.048$). Together, these results confirm that participants' race had no influence in the pattern of results reported in the main analyses.

Supplementary Figure 2

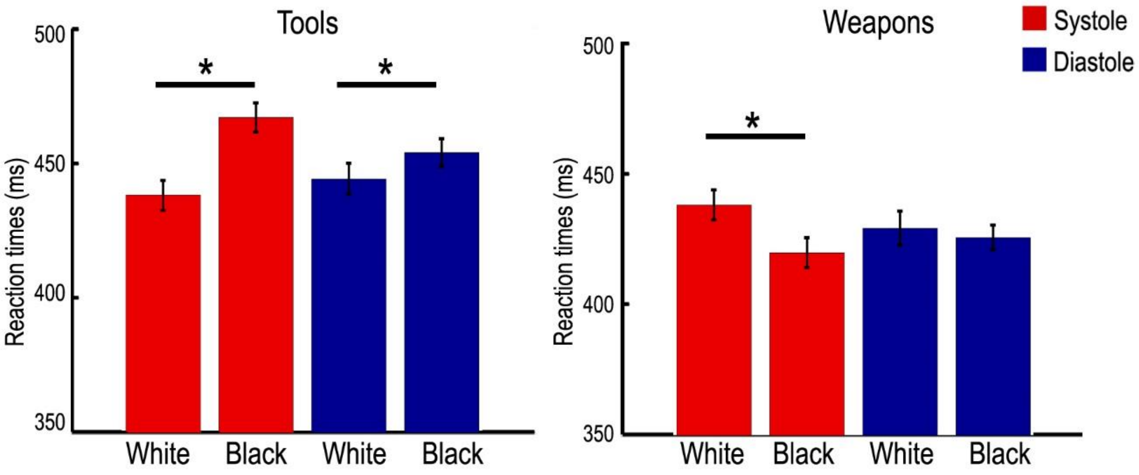

Supplementary Figure 2. Average reaction times (SEM) for correct responses in the WIT **(Study 1)** as a function of race, object type and cardiac cycle. Reaction time analyses were carried out on correct responses occurring between 200-650ms. A Prime (Black/White) x Object (Tool/Weapon) x Cardiac Cycle (Systole/Diastole) repeated measures ANOVA (n=30) revealed similar results to those observed with accuracy rates. The 3-way interaction was significant (F(1,29)=24.21, p<0.001, η^2=0.46) as well as the critical interaction Prime x Object (F(1,29)=43.35, p<0.001 η^2=0.60). Decomposing the interaction according to Object type revealed a Cardiac Cycle x Prime effect on the response times when identifying both Tools (F(1,29)=11.80, p=0.002, η^2=0.29) and Weapons (F(1,29)=7.54, p=0.010, η^2=0.21). The main effect of race was also significant (Tools: F(1,29)= 79.66, p<0.001, η^2=0.73; Weapons: F(1,29)=7.79, p=0.009, η^2=0.21). Planned comparisons (paired t-tests, n=30) confirmed faster identification of tools when preceded by White (vs Black) faces (t(1,29)=8.68, p<0.001, Cohen's d=1.41), and of weapons when preceded by Black (vs White) faces presented at systole (t(1,29)=3.84, p<0.001, Cohen's d=0.70). Conversely, when primes were presented at diastole, participants were only faster identifying tools preceded by White (vs Black) faces (t(1,29)=3.57, p=0.009, Cohen's d=0.54). No difference in reaction times in the identification of weapons was observed (t(1,29)=0.75, p=0.32, Cohen's d=0.14).

Supplementary Figure 3

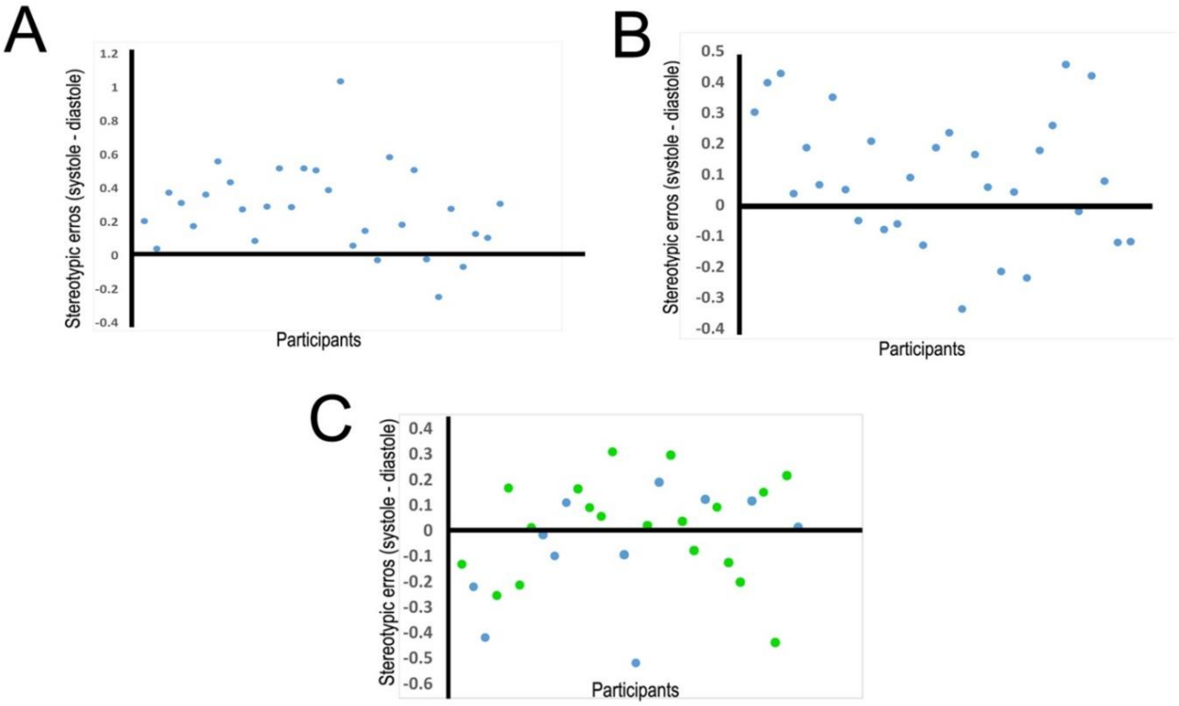

Supplementary Figure 3. A) **Study 1**. Index of stereotypic errors (i.e. misidentification of tools as weapons (vs weapons as tools) after black faces and reduced identification of weapons (vs tools) after white faces) at systole vs diastole for each participant. B) **Study 2**. Index of stereotypic errors (i.e. shooting more often Black “unarmed” than White “unarmed” targets) at systole vs diastole for each participant on “unarmed” trials. C) **Study 3**. Index of stereotypic errors (i.e. misidentification of fruits as sport-objects (vs sport-objects as fruits) after black faces and reduced identification of sport-objects (vs fruits) after white faces) at systole vs diastole for each participant. Green dots – White participants; Blue dots – Asian and mixed-Asian participants. In all three studies, positive values indicate that participants made more stereotypical errors when the prime was presented at systole versus diastole.

Supplementary Figure 4

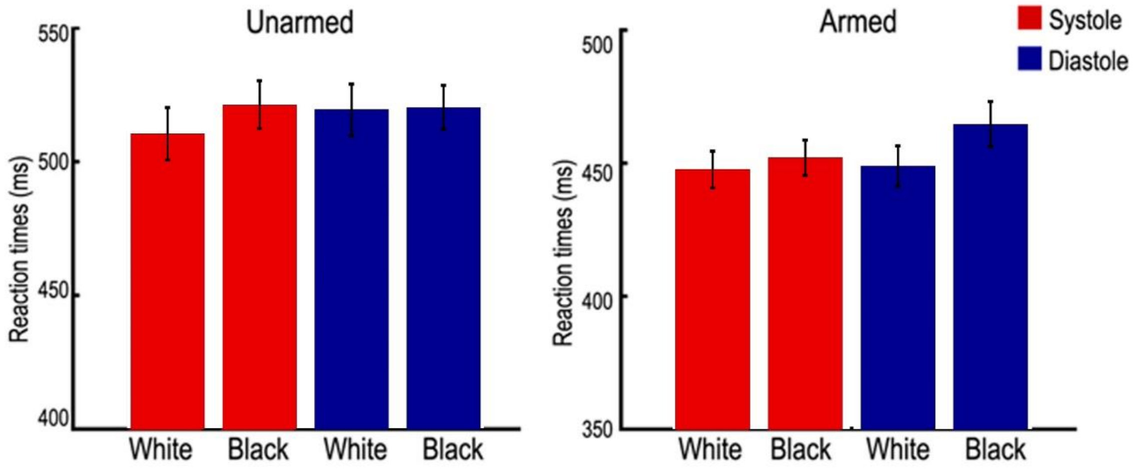

Supplementary Figure 4. Average reaction times (SEM) for correct responses in the FPST **(Study 2)** as a function of race, object type and cardiac cycle. **R**eaction time analyses were carried out on correct responses occurring between 200-1000ms. Average values were entered into an Object (Weapon/No Weapon) x Race (Black/White) x Cardiac Cycle (Systole/Diastole) repeated measures ANOVA (n=30). The interaction was found to be significant (F(1,29)=7.62, p=0.010, η^2=0.21). However, when breaking down the interaction according to Object type, cardiac timing did not interact with race in response times to neither armed (F(1,29)=2.70, p=0.11, η^2=0.08) or unarmed targets (F(1,29)=3.02, p=0.093, η^2=0.09). The fact that we did not find consistent effects in reaction times is not surprising as previous research has shown that bias in reaction times typically occur when participants are allowed longer time to respond and not when they are given shorter time windows forcing them to make mistakes (1). It should be noted that, even if reaction times up to 1000ms were analysed, participants were not only encouraged to respond within 650ms as they received the feedback “Please try to be quicker” and related score penalization whenever their responses exceeded this period. Thus, effectively this study used a 650ms time window and our results are therefore consistent with the pattern of results typically observed in these studies.

Supplementary Figure 5

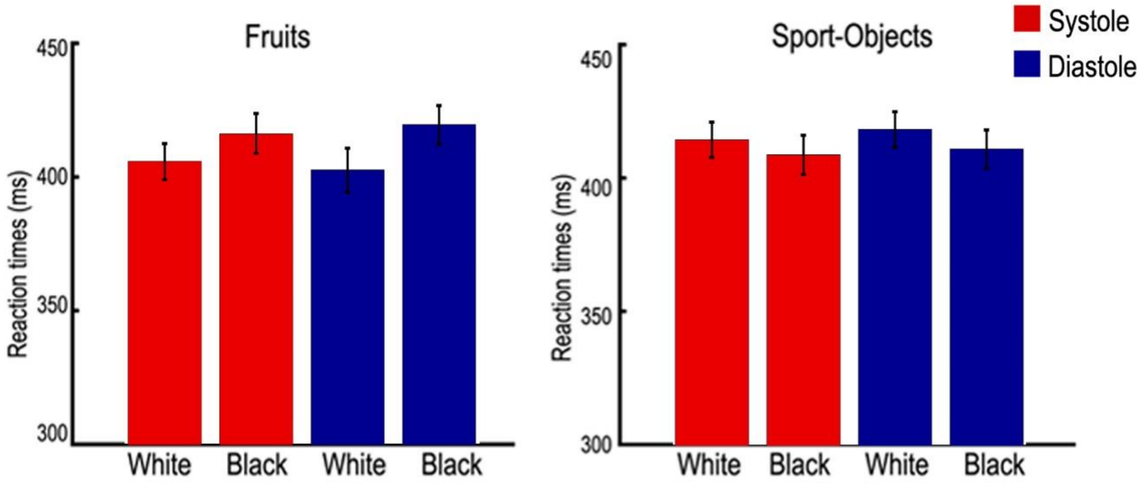

Supplementary Figure 5. Average reaction times (SEM) for correct responses in the SFIT **(Study 3)** as a function of race, object type and cardiac cycle. The Prime (Black/White) x Object (Fruit/Sport-Object) x Cardiac Cycle (Systole/Diastole) repeated measures ANOVA (n=30) on reaction times was not significant ($F(1,29)=1.66$, $p=0.21$, $\eta 2=0.054$) . Separate analyses according to Object type, confirmed that Cardiac Cycle did not modulate race bias in response times to neither Fruits ($F(1,29)=1.77$, $p=1.94$, $\eta 2=0.058$) nor Sport-Objects ($F(1,29)=0.14$, $p=0.71$, $\eta 2<0.01$).

Supplementary note 1

To compare results of Study 3 with those observed in Study 1, we carried out a repeated measures ANOVA (n=60) on accuracy rates with Prime (Black/White) x Object (Fruits or Tools/Sport-Objects or Weapons) x Cardiac Cycle (Systole/Diastole) as within factors and Task (SFIT/WIT) as between participants factor. The 4-way interaction was found to be significant ($F(1,58)=24.87$, $p<0.001$, $\eta^2=0.30$) but not the Prime x Object x Task interaction ($F(1,58)=0.82$, $p=0.37$, $\eta^2=0.014$), confirming that while cardiac cycle did have a differential effect in both tasks the degree of racial bias did not differ across studies. However, we should note that these studies were carried out sequentially and therefore such comparison between studies should be interpreted with the caution.

Supplementary note 2

In Study 1 upon task completion, participants were asked to perform the **heartbeat counting task** (5), a widely used method to measure the individual ability to attend to cardiac signals. In this task, participants should silently count their heartbeats without taking their pulse in four trials of 25, 35, 45 and 100 seconds. The number of counted heartbeats is compared with the number of recorded heartbeats to calculate an index of interoceptive accuracy (IAcc) (5). Values range from 0 – 1 with higher values reflecting greater accuracy. To explore if IAcc is related with cardiac timing effects in the WIT we correlated individual IAcc scores with a Race x Cardiac Cycle index derived from the WIT accuracy rates (see Supplementary Fig. 3A):

(ARcongruent - ARincongruent)Systole - (ARcongruent - ARincongruent)Diastole

where AR stands for accuracy rates, congruent for stereotype congruent trials (Black/Weapon; White/Tool) and incongruent for stereotype incongruent trials (Black/Object; White/Weapon). No relationship between the two variables was found ($r=-0.12$, $p=0.53$). IAcc measures only one of the various dimensions of interoception (6), which is the ability to consciously detect heartbeats at rest, and not much is known yet on how it relates to baroreceptor-related influences. To the best of our knowledge, only one study so far has found that levels of IAcc modulate baroreceptor effects in cognition (7). Thus, while IAcc is likely to mediate cardiac timing effects, at least in certain circumstances, this may not be a linear relationship and be dependent on other psycho-physiological variables.

Supplementary note 3

In study 2, participants also performed a standard version of the **Implicit Association Test (IAT)** (8) (see below), perhaps the most widely used task to measure implicit social cognitions. Unlike the previously described tasks that focus on stereotypes, or specific semantic associations, the IAT evaluates the relative preferences of one social group over another and therefore taps into general attitudes against Black individuals. Performance was analysed according to the improved algorithm proposed by Greenwald and colleagues (8). Positive D scores reflect preference for White over Black individuals. Negative D scores reflect the opposite preference. Results revealed an implicit bias against Black individuals (mean=0.80, s.d.= 0.46). We then carried out correlation analyses between this general index of bias (D) and two indices of Black-threat stereotypes derived from performance in the FPST. One index (FPST-general) reflects the bias to make stereotypic errors independently of cardiac cycle and was calculated with the formula

ARcongruent - ARincongruent

where AR stands for accuracy rates. The other index (FPST-cardiac-cycle) reflects the difference in the tendency to make stereotypic errors in the different phases of the cardiac cycle, and was estimated with the formula (see Supplementary Fig. 3B)

(ARcongruent - ARincongruent)Systole - (ARincongruent - ARincongruent)Diastole

D scores were found to correlate with the FPST-general (r=0.401, p=0.028) but not with the FPST-cardiac-cycle effect (r=0.115, p=0.54). This confirms that, although conceptually different, these two tasks partially overlap. Nevertheless, general attitudes do not seem to explain cardiac timing effects in the FPST that should rely mostly on variables mediating brain-body interactions. We also correlated individual levels of IAcc with the FPST-cardiac-cycle but found no significant correlation (r=-0.090, p=0.64).

Supplementary methods 1

In Study 2, target stimuli were presented for a limited duration of 200ms, in contrast to the longer time-windows (e.g. 630ms) used in this task (2). This had the purpose of ensuring that the stimulus was perceived during specific phases of the cardiac cycle. However, this procedure poses additional demands to the visual processing of these complex images. To make sure that this time window was sufficient for object discrimination, after task completion participants were asked the following forced-choice questions: 1) "was the difficulty of this task related to the need to provide a speeded response or to actually discriminate the object?" 2) "were you able to identify the object even in situations in which you made an error?". **These questions were followed by further questioning, in a semi-structured way, to guarantee that answers to the debriefing questions correctly reflected participants' subjective difficulties in discriminating the objects. Example: "Imagine the following: now the pictures would be presented for the same duration but you would have no time constrain to provide an answer, do you think you would have close to perfect accuracy?"**. Five participants reported difficulties identifying the objects, i.e. answered "discriminate" to the first question and/or "no" to the second question. Their data was excluded from analyses. Additionally, following (2 – study 2), overall error rates for each stimulus were analysed to identify pictures with visual properties that made object discrimination particularly difficult. Pictures with error rates exceeding one-third (26 out of 148, i.e. ~15%) were excluded. It should be noted that for each participant, each stimulus was randomly assigned to the systole and diastole condition and therefore overall error rates for each stimulus reflect differences in visual properties of the picture and not cardiac timing effects.

Implicit Association Test (IAT). In each trial of the IAT, stimuli presented on the centre of the computer screen should be categorized with a key press – "e" for left and "i" for right – according to labels displayed on top left and right corners of the screen, respectively. Stimuli consisted in pictures of 10 White male and 10 Black male individuals' faces, taken from (9), and positive (love, joy, peace, happy, lucky) and negative (ugly, awful, filth, sad, evil) words. Attributes labels were "Good" and

“Bad” and race labels were “White” and “Black”. The task is divided in congruent and incongruent blocks. In congruent trials stimuli are grouped, i.e. share the same response key, according to what are expected to be the easier associations (White/good and Black/bad). In incongruent trials stimuli grouping is made to reflect the opposite associations (White/good and Black/bad) (for further details see (8)). Participants are instructed to answer as fast and as accurately as possible, but no restriction to response times was imposed. Slower reaction times in incongruent trials than in congruent trials are taken to reflect negative implicit bias against Black people. Within each block, stimuli presentation was fully randomized and had no predetermined contingency with the different phases of the cardiac cycle.

References

1 – Payne, B.K. Prejudice and perception: The role of automatic and controlled processes in misperceiving a weapon. *J Pers Soc Psychol* 81, 181-192 (2001).

2– Correll, J., Park, B., Judd, C.M. & Wittenbrink, B. The police officer's dilemma: Using ethnicity to disambiguate potentially threatening individuals. *J Pers Soc Psychol* 83, 1314-1329 (2002).

3 – Krieglmeyer, R. & Sherman, J.W. Disentangling stereotype activation and stereotype application in the stereotype misperception task. *J Pers Soc Psychol* 103(2):205-24 (2012).

4– Judd, C.M., Blair, I.V. & Chapleau, K.M. Automatic stereotypes vs. automatic prejudice: Sorting out the possibilities in the weapon paradigm. *J Exp Soc Psychol* 40 (1), 75– 81 (2004).

5 – Schandry, R. Heart beat perception and emotional experience. *Psychophysiology* 18:483–488 (1981).

6 – Garfinkel, S.N., Seth, A.K., Barrett, A.B., Suzuki, K. & Critchley, H.D. Knowing your own heart: Distinguishing interoceptive accuracy from interoceptive awareness. *Bio Psy* 104, 65-74 (2015).

7 – Garfinkel, S.N., Barrett, A.B., Minati, L., Dolan, R.J., Seth, A.K. & Critchley, H.D. What the heart forgets: Cardiac timing influences memory for words and is modulated by metacognition and interoceptive sensitivity. *Psychophysiology* 50(6):505-12 (2013).

8 – Greenwald, A.G., Nosek, B.A. & Banaji, M.R. Understanding and using the implicit association test: I. An improved scoring algorithm. *J Pers Soc Psychol* 85(2), 197-216 (2003).

9 – Nosek, B.A., Smyth, F.L., Hansen, J.J., Devos, T., Lindner, N.M., Ranganath, K.A., Smith, C.T., Olson, K.R., Chugh, D., Greenwald, A.G. & Banaji, M.R. Pervasiveness and correlates of implicit attitudes and stereotypes. *Eur Rev Soc Psychol* 18, 36-88 (2007).
